# Implementation and Sustainability of Autonomy Initiatives in Long-Term Care Settings

**DOI:** 10.1093/geroni/igaf122.2396

**Published:** 2025-12-31

**Authors:** Ashley Smith, A Lynn Snow, Michelle Hilgeman

**Affiliations:** The University of Alabama, Tuscaloosa, Alabama, United States; The University of Alabama, Tuscaloosa, Alabama, United States; Tuscaloosa VA Medical Center, Tuscaloosa, Alabama, United States

## Abstract

Resident autonomy is a core component of person-centered care in nursing homes yet autonomy-focused interventions face implementation and sustainability challenges (Boumans et al., 2021). The Montessori approach promotes autonomy through individualized choice, engagement, and environmental modifications and is increasingly applied in long-term care settings (Yan & Traynor, 2023). Collective autonomy (e.g., shared decision-making) represents an important but often underexplored aspect of autonomy. This secondary data analysis examines changes in resident autonomy during implementation of Montessori staff training in a cluster randomized study in 8 US Veterans Health Administration Community Living Centers (VA nursing homes). The Montessori Organizational Self-Assessment (MOST) was used to assess four autonomy domains across 5 timepoints: 1) Ask the Resident, 2) Connecting with Others, 3) Prepare the Environment, and 4) Create Community, rated on a scale from 0 (Not There) to 2 (In Action). At baseline, sites rated themselves below the midpoint in all domains, with the highest scores in Domain 1 (M = .88, SD = .07) and Domain 3 (M = .82, SD = .10). Linear mixed-effects models showed significant increases from baseline to 12 months across all domains. However, only Domain 1 demonstrated sustained improvement at each follow-up. Other domains improved by 12 months but showed variability across intermediate timepoints. To our knowledge, this is one of the first studies to examine MOST scores as an indicator of collective autonomy over time after implementation of a complex intervention. Findings suggest collective autonomy progress may be challenging to sustain. Future research should investigate strategies to reinforce autonomy across all domains and the relationship between environmental and process-related autonomy initiatives.

